# Clinical Features, Management, and Molecular Characteristics of Familial Small Bowel Neuroendocrine Tumors

**DOI:** 10.3389/fendo.2021.622693

**Published:** 2021-02-26

**Authors:** James Y. Lim, Rodney F. Pommier

**Affiliations:** Division of Surgical Oncology, Department of Surgery, Oregon Health & Science University, Portland, OR, United States

**Keywords:** carcinoid, familial, small bowel neuroendocrine tumors, molecular characteristics of the tumor, clinical management

## Abstract

Small bowel neuroendocrine tumors are rare tumors with an increasing incidence over the last several decades. Early detection remains challenging because patients commonly develop symptoms late in the disease course, often after the tumors have metastasized. Although these tumors were thought to arise from sporadic genetic mutations, large epidemiological studies strongly support genetic predisposition and increased risk of disease in affected families. Recent studies of familial small bowel neuroendocrine tumors have identified several novel genetic mutations. Screening for familial small bowel neuroendocrine tumors can lead to earlier diagnosis and improved patient outcomes. This review aims to summarize the current knowledge of molecular changes seen in familial small bowel neuroendocrine tumors, identify clinical features specific to familial disease, and provide strategies for screening and treatment.

## Introduction

Neuroendocrine tumors were once considered uncommon tumors; however, the age-adjusted incidence rates have steadily increased by 6.4 fold from 1973 to 2012 (1). Small bowel neuroendocrine tumors (SBNETs) are second to the lung as the most common primary site of disease with an annual incidence of 1.05 per 100,000 (1).

SBNET are generally indolent tumors, but these are also the most common neuroendocrine tumors to metastasize such that 60–80% of patients with SBNET have liver metastases on initial presentation (2, 3). The delay in diagnosis is primarily due to vague, non-specific symptoms that are easily attributed to other benign conditions (4). Symptoms of obstruction or carcinoid syndrome develop only after the disease has metastasized to adjacent lymph nodes or other organs (3). Because widely metastatic disease is currently incurable, methods to identify asymptomatic patients are of paramount importance.

SBNET were previously thought to be rare sporadic tumors; however familial SBNET (defined as two cases in two first degree relatives with no other causative genetic syndrome) have been reported since the 1960s (5–7). In addition to case series, larger national epidemiological studies looking at neuroendocrine tumors have also looked at family associations and confirmed increased risks within familial cohorts (5, 6, 8, 9). These larger studies showed a relative risk of 2.8 to 4.3 for the development of neuroendocrine tumors in familial cohorts (8, 10, 11). One study using the national cancer data sets of Sweden and Finland, found a 30-fold increased risk of a SBNET among siblings with known SBNET. These data suggest an underlying genetic cause that if identified could facilitate screening of asymptomatic relatives.

Despite this strong epidemiological evidence for a genetic predisposition to SBNET in affected families, no single causative mutation has been identified. Instead, several pathways have been implicated. This review will describe the various mutations that have been identified and validated for sporadic and familial SBNET. We will also review the clinical features and management of patients with and at risk for familial SBNET.

## Molecular Characteristics of Sporadic and Familial Small Bowel Net

### Sporadic SBNET

The genomic landscape of sporadic SBNET has been investigated in order to identify drivers for tumorigenesis and targets for treatment based on molecular profiling. Overall, SBNETs have been found to be mutationally silent tumors with low overall mutation rates as compared to other malignancies (12–14). One of the first genome wide sequencing performed on sporadic SBNET showed that there were relatively few somatic mutations, and the most common abnormality identified were somatic cell number alterations. These alterations specifically implicated PI3K/Akt/mTOR signaling, and the TGF-*β* pathway through alterations in SMAD genes (14). However, another study looking at mutational analysis of exome sequencing of 55 SBNET identified 1,230 somatic mutations, only 21 of which had been previously identified in SBNET studies (15). Of these mutated genes, one of the most frequently mutated was the cell cycle regulatory gene Cyclin Dependent Kinase Inhibitor 1B (*CDKN1B* or p27), and this gene was mutated in up to 5–8% of SBNET (13, 15). Interestingly, mutations of *CDKN1B* are also known as multiple endocrine neoplasia type 4 (MEN4). A recent review showed that MEN4 is associated with parathyroid and pituitary disease primarily. The prevalence of gastrointestinal neuroendocrine tumors is less in MEN4 than in multiple endocrine neoplasia type 1 (MEN1) (16). In addition to CDKN1B, analysis of SBNET samples from the RADIANT trials found recurrent mutations in BCL6 Corepressor (*BCOR*) identified in 5.6% of SBNET (13). Both these somatic mutations were primarily loss-of-function due to frameshifts (13). Another large genome wide association study was performed looking at 293 sporadic SBNET. Three associated single nucleotide polymorphisms were identified on chromosome 12 just upstream of *ELK3*, a transcription factor associated with angiogenesis (17). Earlier studies also identified *APC* and *SRC* mutations in 23 and 25% of SBNET although these have not been confirmed in more recent studies (14, 18).

Aside from genetic mutations, chromosomal analysis has also shown broad recurrent chromosomal aberrations. Loss of heterozygosity in chromosome 18 has been identified in up to 50% of SBNET (12, 13, 19, 20). This frequent alteration has led to further investigation of tumor suppressor genes contained within chromosome 18 with conflicting conclusions. Nieser et al. performed expression analysis on putative tumor suppressors found on chromosome 18 in SBNET and concluded that there were no significant changes in expression of tumor suppressor genes *SMAD2, SMAD4*, and *TCEB3C/Elongin A3.* They did identify a reduced expression of *DCC* (deleted in colorectal cancer) in 29% of cases (21). More recently, Roland et al. examined 38 neuroendocrine tumors, of which 22 were SBNET, and showed that 29% of the tumors showed loss of *SMAD4* on immunohistochemistry and this finding was associated with poor prognosis after resection (22). Another study by Edfeldt et al. focusing on *TCEB3C*, found that 76.7% of 43 SBNET samples showed significant reduction in expression of elongin A3. Further investigation identified that 89% of the tumors had only one of two copies of the *TCEB3C* gene. *TCEB3C* is unique in that it is the only known imprinted gene on chromosome 18 and, as a potential tumor suppressor gene, could be more vulnerable to a single gene mutation than a non-imprinted gene (23). Survival analysis with these various loss of function mutations has also yielded conflicting results (12). Looking at the above studies, one can see that the only common finding is that there is frequently a loss of heterozygosity in chromosome 18 in SBNET, but the specific gene alteration is not consistent across studies. Furthering this point, a recent study investigating multifocal disease in sporadic SBNET patients showed that primary tumors from the same patient could present with different distinct patterns of chromosome 18 allelic loss (24). Additional chromosomal alterations identified in SBNET have included copy number gains in chromosomes 4, 5, 14, and 20 (12, 13). These chromosomal gains have also had unclear clinical significance with some studies finding an association with worse prognosis, while other studies have not seen any difference. All these different study findings likely point to chromosomal instability as being a common feature of SBNET. When looking at generalized chromosomal instability in SBNET patients (as measured by total copy number aberrations), increased chromosomal instability was associated with poor prognosis in patients. Progression free survival in patients with low chromosomal instability was 18.6 *vs* 9.2 months in patients with high chromosomal instability (*P* = 0.0021) (13).

Epigenetic modifications have also commonly been identified in SBNET. Alterations in DNA methylation were identified in 65–82% of SBNET (12). SBNET epigenetic studies have most commonly shown hypermethylation in chromosome 18 gene sets and also in the promoter region of the gastric inhibitory polypeptide receptor. Tumors found to have this hypermethylation have been associated with more malignant behavior of the SBNET (3, 25). Additional genes that have been identified in SBNET studies to be hypermethylated include *RASSF1A*, *CTNNB1, MGMT*, and *ElonginA3* (23, 26, 27). Genes that have been associated with hypomethylation include *LINE1*, *ALU*, and *UCHL1* (28, 29). Studies comparing primary SBNETs to their matched metastatic tumors have shown significantly different methylation patterns between the tumors (25, 30). It is still unclear how these methylation patterns promote tumorigenesis and progression to metastasis in SBNET.

Studies investigating the role of miRNA in SBNET have largely encompassed comparison of primary tumor profiles to their metastases. There have been several candidate miRNAs that have shown consistent expression patterns across studies, such as miR-133a (31). Overall, epigenetic inheritance in SBNET has not been investigated.

### Familial SBNET

Because of the limited knowledge of molecular characteristics in sporadic SBNET, identifying a molecular driver in familial SBNET has been difficult. Sei et al. describe one of the most recently identified recurring mutations in familial SBNET patients. They describe a four base pair mutation in the inositol polyphosphate multikinase gene (*IPMK*). Whole exome sequencing was performed on germline DNA of the affected family members in one family and the *IPMK* mutation was identified in all 11 known members of the family with SBNET. There were no other candidate genetic mutations identified within the affected family. The hypothesis for pathogenesis with an *IPMK* mutation is a reduction in inositol phosphate kinase function leading to diminished p53 activated apoptosis and increased survival of the tumor cells (32). Interestingly, within this study that identified this mutation, there were 32 additional families classified to have familial SBNET, but none of them were identified to have this mutation. No other candidate gene mutations were identified in the other families. Subsequent studies have also not identified this *IPMK* mutation as a driving mutation in other familial SBNET candidate families (7, 17, 33).

Another study looking at 15 families classified as having familial SBNET in Sweden used next generation sequencing of exome and whole genome sequencing of blood DNA to identify seven different candidate gene mutations: *TERT*, *SDHA, SDHB, SDHD, MUTYH* and *OGG1*. These were all monoallelic germline mutations. Of these mutations, functional mutation damage predicting algorithms were used to classify the significance of these mutations. Mutations in *MUTYH* and *OGG1* were predicted to be the most damaging to human proteins. Both these genes are involved in DNA base excision repair (7). Because of the predictive algorithms, these two genes were then studied in 215 sporadic cases of SBNET, and researchers found an increased frequency of *MUTYH* and *OGG1* mutations in these tumors as well with an odds ratio of 5.09 (7). A recent case report of a young patient known to have *MUTYH* adenomatous polyposis syndrome due to biallelic *MUTYH* mutations and new SBNET diagnosis supports this proposed genetic link (34). Overall, these findings suggest a potential driving role of *MUTYH* and *OGG1* mutations in the development of SBNET.

Comparing chromosomal abnormalities between sporadic and familial SBNET, abnormalities in chromosome 18 were the dominant finding in both groups (35). Findings of deletions in chromosome 18 in familial SBNET have been identified in several studies (33, 35). In one study comparing 37 sporadic cases to eight familial cases, aberrations in chromosome 18 were found in 100% of sporadic cases and 38% of familial cases (35). When gene expression profiles were compared between the two groups though, their profiles were similar with no significant differences. In another series de Mestier et al. identified a chromosome 18 deletion in 80% of patients with familial SBNET. These findings imply that mechanisms for tumorigenesis in SBNET may be more similar between familial and sporadic SBNET.

The molecular landscape of familial SBNET continues to remain sparse despite epidemiological evidence pointing to a significant hereditary risk. Investigations into epigenetic inheritance in familial SBNET may be revealing. Although the *IPMK* mutation appears to be a significant driver in SBNET development in one family; no subsequent familial SBNET studies have identified this mutation in other family cohorts.

## Clinical Features

Familial SBNET is most commonly defined as presence of disease in two first degree relatives without another genetic syndrome. A European study looking at nine different family pedigrees of familial SBNET suggested an autosomal dominant pattern of inheritance with incomplete penetrance. More recently, a US study of 33 SBNET family pedigrees also suggested an autosomal dominant transmission with late onset and incomplete penetrance (36).

There are several clinical features of familial SBNET that differ from sporadic disease. Patients are often diagnosed at a younger age with familial SBNET. Familial SBNET patients were diagnosed at a median age of 57 compared to 61 with sporadic SBNET (7). An earlier age at diagnosis is a characteristic typically seen when there is an inherited component of disease. It is unclear though, given the complicated genetic landscape, if this is due to increased surveillance in family members or if the degree of penetrance leads to earlier disease presentation. This is also complicated by the fact that the natural history of SBNET is a small primary tumor that grows in an indolent fashion with symptoms leading to diagnosis typically occurring with more advanced disease (37). In another study looking at familial SBNET, they found no difference in age of presentation in symptomatic family members, compared to sporadic disease patients. When asymptomatic relatives were screened in the study, they did find that average age of diagnosis for occult disease was younger, 58 *vs* 61, although the difference was not statistically significant (32).

Another difference between familial and sporadic SBNET is the frequent finding of multiple synchronous primary tumors in familial cases. In sporadic cases up to 25–50% of patients may have multiple primary tumors (37, 38). Hughes et al. screened 129 asymptomatic patients from 13 families, and identified 29 patients with occult familial SBNET. After surgical resection of their disease, 24 of the 29 (83%) patients were found to have multifocal disease. The average number of tumors resected was four (range of 1–29), with the majority located in the ileum (55%) followed by the jejunum (24.1%) (36).

Because symptoms of SBNET such as abdominal pain, obstruction and carcinoid syndrome are usually reflective of more advanced disease, the difference in symptoms of patients with familial and sporadic SBNET will depend on the stage at diagnosis. There are no data to suggest that familial SBNET is more aggressive or portends a worse prognosis than sporadic SBNET. One thing to consider is that if relatives of patients with familial SBNET are being proactively screened, they will likely be diagnosed while asymptomatic. In the previous study, where 129 asymptomatic relatives were screened for disease, there was a significant difference in the stage at diagnosis for the 29 asymptomatic patients identified to have SBNET after screening. Stage IV disease was identified in only 8.7% of these patients with occult disease as compared to 70% in their symptomatic relatives (p < 0.001) (36).

In terms of other clinical characteristics, familial SBNET is otherwise indistinguishable from sporadic disease (32, 35). No studies have evaluated the differences in tumor markers between familial and sporadic disease. 24-h urine 5-HIAA, blood serotonin, plasma 5-HIAA, and chromogranin A are frequently used tumor markers for SBNET (37, 39). Previous studies have shown that chromogranin A has a limited sensitivity of 70–90% for diagnosing neuroendocrine tumors and a specificity of 50–67% depending on which laboratory cutoff values were used (39, 40). 24-hour urine 5-HIAA, usually found in those with liver involvement, is associated with a specificity of 88% (39). These laboratory tests require a careful investigation of potential interfering medications and foods prior to obtaining.

SBNET will frequently metastasize to the mesentery, liver, peritoneum and ovaries, so imaging modalities should focus on the abdomen and pelvis (37, 41). Typically, imaging will begin with a chest x-ray and cross-sectional imaging of the abdomen and pelvis with either computed tomography (CT) scan or magnetic resonance imaging (MRI). Imaging should be contrast enhanced with arterial and delayed portal venous phases when looking for metastases as SBNET liver metastases will enhance during the arterial phase and wash out during the venous phase (37, 39). SBNET will also often metastasize to the mesenteric lymph nodes that are classically seen in the root of the mesentery with a radial pattern of fibrosis due to the desmoplastic reaction. The primary tumors are often small and are not able to be seen on these standard imaging modalities. Other imaging modalities that can be used with varying success for primary tumor identification include double balloon enteroscopy, video capsule endoscopy, and CT enterography (37, 38, 42). Video capsule endoscopy is contraindicated in patients with impending bowel obstruction (43). When preoperative evaluation is unable to identify the primary tumor, studies have shown that the primary tumor can be identified at surgical exploration with a high rate of success (44, 45).

Imaging modalities can also take advantage of the fact that most SBNET express somatostatin receptors and there are currently several somatostatin receptor scintigraphy options. The first approved option used the somatostatin analogue octreotide radiolabeled with indium-111 coupled today with single photon emission computed tomography (SPECT)/CT. More recently, higher affinity somatostatin analogs, such as tyrosine-3-octreotate are linked *via* dodecanetetraacetic acid (DOTATATE) with positron emitting radio-isotopes such as 68-gallium in conjunction with positron emitting tomography (PET)/CT in order to provide images with improved resolution and dosimetry. In an asymptomatic familial SBNET patient with a known genetic mutation, somatostatin receptor scintigraphy may be the most sensitive method of detecting localized disease. There have been several studies showing that somatostatin receptor PET imaging is able to identify primary tumors with an increased sensitivity as compared to other imaging modalities (46, 47). Additional means of establishing a SBNET diagnosis includes biopsy either of the primary tumor or metastases.

## Management

Currently, there are no standardized screening guidelines for asymptomatic relatives of patients with familial SBNET. There are recommendations for patients at high risk for neuroendocrine tumors of the foregut such as in MEN1 patients, and some parallels can be drawn to familial SBNET patients. Screening recommendations also depend on whether patients have an identifiable genetic mutation. The *IPMK* mutation appears to be the best described with a clear pattern of inheritance in one family, but unfortunately this mutation has not been identified in any other families. Until the genetic basis of familial SBNET becomes clearer, all relatives in families with suspected familial SBNET should undergo interval surveillance workup for SBNET with labs and cross-sectional imaging as discussed previously. Because asymptomatic individuals are more likely to have early stage vs. late stage disease, CT enterography and/or somatostatin receptor scintigraphy may offer increased sensitivity in identifying a primary tumor. Because SBNETs are typically slow growing tumors, if the initial screening workup is negative, surveillance workup can likely be safely extended to a 2–3 year interval. The National Institute of Health’s screening protocol in their familial SBNET study was to screen asymptomatic relatives every 2 years (36).

If localized disease is identified, international guidelines recommend treatment consisting of surgical resection of the affected small bowel and its associated regional lymph nodes following oncological principles (3, 39, 43, 48). Complete resection of locoregional disease can be achieved in up to 80% of patients treated at experienced centers (49). Resectability is determined by the location and extent of the mesenteric disease and its associated desmoplastic reaction. The exact number of lymph nodes that determines an adequate lymphadenectomy is controversial. In addition, it may be difficult to count the exact number of lymph nodes contained within a large mesenteric nodal mass. A recent multi-institutional study did conclude that accurate lymph node staging needed a minimum of eight lymph nodes and having four or more involved lymph nodes was associated with a decreased 3-year recurrence free survival (50).

Current recommendations by the North American Neuroendocrine Tumor Society (NANETS) are to perform an open operation in order to be able to carefully palpate and examine the entire length of small bowel for occult tumors (3, 44). A purely laparoscopic operation is considered inadequate for running the bowel and any laparoscopic approach should be balanced by the principles of oncologic resection. In particular for patients with familial SBNET, an open operation seems prudent given the high likelihood of multiple synchronous tumors. In addition, an intraoperative ultrasonography of the liver can be performed at the same time to screen for occult liver metastases (3).

In patients with metastatic disease, aggressive operative resection can be considered in good surgical candidates for optimal patient outcomes. The liver is the most common site of metastasis and subsequent liver failure secondary to metastases is the most common cause of death. Metastatic liver disease can be cytoreduced using parenchyma sparing techniques if at least a 70% debulking threshold can be achieved (2, 3, 51). In patients with liver disease, patients should be evaluated preoperatively for carcinoid heart disease. If present, the heart valves will require repair prior to oncologic resection (39, 52). Cholecystectomy should be performed in cases of metastatic disease given the high likelihood of requiring long term somatostatin analog treatment which can induce cholelithiasis that may become symptomatic and/or complicated (3, 52). In addition, resection of the primary tumor should be considered even in the setting of inoperable disease as studies have shown that resection of a primary SBNET is associated with, and in some studies, an independent predictor for improved survival (53–57). A recent systematic review and meta-analysis looking at primary tumor resection in the setting of inoperable liver metastases showed a pooled overall survival of 73.1% in the primary tumor resection group as compared to 36.6% in the non-resection group (58).

During operations in patients with metastatic disease, the operative team needs to be aware of the possibility of carcinoid crises. Intra-operative carcinoid crises are typically characterized by severe hypotension and can include bronchospasm and flushing. It has long been thought to be due to the release of massive amounts of vasoactive substances from the tumor. Prospective investigation of perioperative hormone levels only identified a high preoperative serotonin level as being predictive of a carcinoid crisis occurrence (59), but failed to show increased levels of serotonin, histamine, bradykinin, or kallikrein during crises compared to pre-incision levels. Although crises occur most frequently in patients with SBNET metastatic to the liver, it can occur also in patients with retroperitoneal or ovarian metastases (43).

Beyond an operation, oncologic treatment for metastatic disease includes somatostatin analogues, local liver directed therapies, chemotherapy and peptide receptor radionuclide therapy (PRRT) (37, 39, 52). Somatostatin analogues can control symptoms of carcinoid syndrome and have also been shown to inhibit tumor growth in randomized trials (60, 61). Although standard cytotoxic chemotherapy is reserved for poorly differentiated SBNET, newer agents such as antiangiogenesis drugs and mammalian target of rapamycin inhibitors have shown improved progression free survival in clinical trials (62). Finally, PRRT is a recently approved systemic treatment using radiolabeled (lutetium-177) octreotide that also has shown improved progression free survival and radiographic response as compared to the control high dose somatostatin analogue cohort. In the NETTER-1 trial, the progression free-survival rate at 20 months in the PRRT group was 65.2% as compared to 10.8% in the control group (63). More studies of this promising treatment are needed, as neuroendocrine tumors of different primaries may respond differently to PRRT (64). Utilization of these different therapies should involve a multidisciplinary team that understands the benefits and the risks of the various treatment options. Follow-up of patients after either surgical resection or medical treatment should continue with both imaging and labwork in 6–12 month intervals (3, 43, 48, 65). In the setting of a familial SBNET, the surveillance should be lifelong given the underlying germline mutation that is suspected to be involved.

## Conclusion

Familial SBNET is a recently identified entity that still needs further investigation. Several distinct mutations have been described. The benefits of screening for familial SBNET are apparent when comparing the stage of diagnosis in asymptomatic *vs* symptomatic patients. With the future identification of pathologic mutations in familial SBNET, highly effective screening can be limited to those members of the family who are at risk.

## Author Contributions

JL conceived the project and wrote the manuscript. RP provided critical evaluation of the manuscript and scientific content. All authors contributed to the article and approved the submitted version.

## Conflict of Interest

The authors declare that the research was conducted in the absence of any commercial or financial relationships that could be construed as a potential conflict of interest.

## References

[1] Dasari A Shen C Halperin D Zhao B Zhou S Xu Y. Trends in the Incidence, Prevalence, and Survival Outcomes in Patients With Neuroendocrine Tumors in the United States. JAMA Oncol (2017) 3(10):1335–42. 10.1001/jamaoncol.2017.0589 PMC5824320 28448665

[2] Graff-Baker AN Sauer DA Pommier SJ Pommier RF. Expanded criteria for carcinoid liver debulking: Maintaining survival and increasing the number of eligible patients. Surgery (2014) 156(6):1369–76; discussion 76-7. 10.1016/j.surg.2014.08.009 25456912

[3] Howe JR Cardona K Fraker DL Kebebew E Untch BR Wang YZ. The Surgical Management of Small Bowel Neuroendocrine Tumors: Consensus Guidelines of the North American Neuroendocrine Tumor Society. Pancreas (2017) 46(6):715–31. 10.1097/MPA.0000000000000846 PMC5502737 28609357

[4] Toth-Fejel S Pommier RF. Relationships among delay of diagnosis, extent of disease, and survival in patients with abdominal carcinoid tumors. Am J Surg (2004) 187(5):575–9. 10.1016/j.amjsurg.2004.01.019 15135668

[5] Pal T Liede A Mitchell M Calender A Narod SA. Intestinal carcinoid tumours in a father and daughter. Can J Gastroenterol = J Canadien Gastroenterologie (2001) 15(6):405–9. 10.1155/2001/908056 11429670

[6] Babovic-Vuksanovic D Constantinou CL Rubin J Rowland CM Schaid DJ Karnes PS. Familial occurrence of carcinoid tumors and association with other malignant neoplasms. Cancer Epidemiol Biomarkers Prev Publ Am Assoc Cancer Research Cosponsored by Am Soc Prev Oncol (1999) 8(8):715–9. 10.1530/ERC-16-0171 10744132

[7] Dumanski JP Rasi C Bjorklund P Davies H Ali AS Gronberg M. A MUTYH germline mutation is associated with small intestinal neuroendocrine tumors. Endocrine Related Cancer (2017) 24(8):427–43. 10.1530/ERC-17-0196 PMC5527373 28634180

[8] Hiripi E Bermejo JL Sundquist J Hemminki K. Familial gastrointestinal carcinoid tumours and associated cancers. Ann Oncol Off J Eur Soc Med Oncol (2009) 20(5):950–4. 10.1093/annonc/mdn706 19150948

[9] Kharazmi E Pukkala E Sundquist K Hemminki K. Familial risk of small intestinal carcinoid and adenocarcinoma. Clin Gastroenterol Hepatol Off Clin Pract J Am Gastroenterol Assoc (2013) 11(8):944–9. 10.1016/j.cgh.2013.02.025 23500615

[10] Hemminki K Li X. Incidence trends and risk factors of carcinoid tumors: a nationwide epidemiologic study from Sweden. Cancer (2001) 92(8):2204–10. 10.1002/1097-0142(20011015)92:8<2204::aid-cncr1564>3.0.co;2-r 11596039

[11] Neklason DW VanDerslice J Curtin K Cannon-Albright LA. Evidence for a heritable contribution to neuroendocrine tumors of the small intestine. Endocrine Related Cancer (2016) 23(2):93–100. 10.1530/ERC-15-0442 26604321 PMC4684974

[12] Samsom KG van Veenendaal LM Valk GD Vriens MR Tesselaar MET van den Berg JG. Molecular prognostic factors in small-intestinal neuroendocrine tumours. Endocrine Connections (2019) 8(7):906–22. 10.1530/EC-19-0206 PMC6599083 31189127

[13] Yao J Garg A Chen D Capdevila J Engstrom P Pommier R. Genomic profiling of NETs: a comprehensive analysis of the RADIANT trials. Endocrine Related Cancer (2019) 26(4):391–403. 10.1530/ERC-18-0332 30667365

[14] Banck MS Kanwar R Kulkarni AA Boora GK Metge F Kipp BR. The genomic landscape of small intestine neuroendocrine tumors. J Clin Invest (2013) 123(6):2502–8. 10.1172/JCI67963 PMC3668835 23676460

[15] Francis JM Kiezun A Ramos AH Serra S Pedamallu CS Qian ZR. Somatic mutation of CDKN1B in small intestine neuroendocrine tumors. Nat Genet (2013) 45(12):1483–6. 10.1038/ng.2821 PMC4239432 24185511

[16] Frederiksen A Rossing M Hermann P Ejersted C Thakker RV Frost M. Clinical Features of Multiple Endocrine Neoplasia Type 4: Novel Pathogenic Variant and Review of Published Cases. J Clin Endocrinol Metab (2019) 104(9):3637–46. 10.1210/jc.2019-00082 PMC6637788 30990521

[17] Du Y Ter-Minassian M Brais L Brooks N Waldron A Chan JA. Genetic associations with neuroendocrine tumor risk: results from a genome-wide association study. Endocrine Related Cancer (2016) 23(8):587–94. 10.1530/ERC-16-0171 PMC6151867 27492634

[18] Bottarelli L Azzoni C Pizzi S D’Adda T Silini EM Bordi C. Adenomatous polyposis coli gene involvement in ileal enterochromaffin cell neuroendocrine neoplasms. Hum Pathol (2013) 44(12):2736–42. 10.1016/j.humpath.2013.06.019 24139208

[19] Mafficini A Scarpa A. Genetics and Epigenetics of Gastroenteropancreatic Neuroendocrine Neoplasms. Endocrine Rev (2019) 40(2):506–36. 10.1210/er.2018-00160 PMC6534496 30657883

[20] Wang GG Yao JC Worah S White JA Luna R Wu TT. Comparison of genetic alterations in neuroendocrine tumors: frequent loss of chromosome 18 in ileal carcinoid tumors. Modern Pathol (2005) 18(8):1079–87. 10.1038/modpathol.3800389 15920555

[21] Nieser M Henopp T Brix J Stoss L Sitek B Naboulsi W. Loss of Chromosome 18 in Neuroendocrine Tumors of the Small Intestine: The Enigma Remains. Neuroendocrinology (2017) 104(3):302–12. 10.1159/000446917 27222126

[22] Roland CL Starker LF Kang Y Chatterjee D Estrella J Rashid A. Loss of DPC4/SMAD4 expression in primary gastrointestinal neuroendocrine tumors is associated with cancer-related death after resection. Surgery (2017) 161(3):753–9. 10.1016/j.surg.2016.09.002 PMC5937257 27816207

[23] Edfeldt K Ahmad T Akerstrom G Janson ET Hellman P Stalberg P. TCEB3C a putative tumor suppressor gene of small intestinal neuroendocrine tumors. Endocrine Related Cancer (2014) 21(2):275–84. 10.1530/ERC-13-0419 24351681

[24] Zhang Z Makinen N Kasai Y Kim GE Diosdado B Nakakura E. Patterns of chromosome 18 loss of heterozygosity in multifocal ileal neuroendocrine tumors. Genes Chromosomes Cancer (2020) 59(9):535–9. 10.1002/gcc.22850 PMC7384092 32291827

[25] Verdugo AD Crona J Starker L Stalberg P Akerstrom G Westin G. Global DNA methylation patterns through an array-based approach in small intestinal neuroendocrine tumors. Endocrine Related Cancer (2014) 21(1):L5–7. 10.1530/ERC-13-0481 24192231

[26] Zhang HY Rumilla KM Jin L Nakamura N Stilling GA Ruebel KH. Association of DNA methylation and epigenetic inactivation of RASSF1A and beta-catenin with metastasis in small bowel carcinoid tumors. Endocrine (2006) 30(3):299–306. 10.1007/s12020-006-0008-1 17526942

[27] Finnerty BM Gray KD Moore MD Zarnegar R Fahey Iii TJ. Epigenetics of gastroenteropancreatic neuroendocrine tumors: A clinicopathologic perspective. World J Gastrointest Oncol (2017) 9(9):341–53. 10.4251/wjgo.v9.i9.341 PMC5605334 28979716

[28] Kleiman DA Beninato T Sultan S Crowley MJ Finnerty B Kumar R. Silencing of UCHL1 by CpG promoter hyper-methylation is associated with metastatic gastroenteropancreatic well-differentiated neuroendocrine (carcinoid) tumors. Ann Surg Oncol (2014) 21(Suppl 4):S672–9. 10.1245/s10434-014-3787-2 PMC4378537 24854489

[29] Choi IS Estecio MR Nagano Y Kim DH White JA Yao JC. Hypomethylation of LINE-1 and Alu in well-differentiated neuroendocrine tumors (pancreatic endocrine tumors and carcinoid tumors). Modern Pathol an Off J U States Can Acad Pathol Inc (2007) 20(7):802–10. 10.1038/modpathol.3800825 17483816

[30] Karpathakis A Dibra H Pipinikas C Feber A Morris T Francis J. Progressive epigenetic dysregulation in neuroendocrine tumour liver metastases. Endocrine Related Cancer (2017) 24(2):L21–L5. 10.1530/ERC-16-0419 28049633

[31] Miller HC Frampton AE Malczewska A Ottaviani S Stronach EA Flora R. MicroRNAs associated with small bowel neuroendocrine tumours and their metastases. Endocrine Related Cancer (2016) 23(9):711–26. 10.1530/ERC-16-0044 27353039

[32] Sei Y Zhao X Forbes J Szymczak S Li Q Trivedi A. A Hereditary Form of Small Intestinal Carcinoid Associated With a Germline Mutation in Inositol Polyphosphate Multikinase. Gastroenterology (2015) 149(1):67–78. 10.1053/j.gastro.2015.04.008 25865046 PMC4858647

[33] de Mestier L Pasmant E Fleury C Brixi H Sohier P Feron T. Familial small-intestine carcinoids: Chromosomal alterations and germline inositol polyphosphate multikinase sequencing. Digest liver Dis Off J Ital Soc Gastroenterol Ital Assoc Study Liver (2017) 49(1):98–102. 10.1016/j.dld.2016.10.007 27825921

[34] Weidner TK Kidwell JT Glasgow AE Menias CO Ahn DH Pai RK. Small Intestine Neuroendocrine Tumor in a Patient With MUTYH Adenomatous Polyposis-Case Report and SEER Analysis. Clin Colorectal Cancer (2018) 17(3):e545–e8. 10.1016/j.clcc.2018.05.002 29843990

[35] Cunningham JL Diaz de Stahl T Sjoblom T Westin G Dumanski JP Janson ET. Common pathogenetic mechanism involving human chromosome 18 in familial and sporadic ileal carcinoid tumors. Genes Chromosomes Cancer (2011) 50(2):82–94. 10.1002/gcc.20834 21104784

[36] Hughes MS Azoury SC Assadipour Y Straughan DM Trivedi AN Lim RM. Prospective evaluation and treatment of familial carcinoid small intestine neuroendocrine tumors (SI-NETs). Surgery (2016) 159(1):350–6. 10.1016/j.surg.2015.05.041 PMC4858646 26454678

[37] Strosberg J. Neuroendocrine tumours of the small intestine. Best Pract Res Clin Gastroenterol (2012) 26(6):755–73. 10.1016/j.bpg.2012.12.002 23582917

[38] Gangi A Siegel E Barmparas G Lo S Jamil LH Hendifar A. Multifocality in Small Bowel Neuroendocrine Tumors. J Gastrointest Surg Off J Soc Surg Aliment Tract (2018) 22(2):303–9. 10.1007/s11605-017-3586-8 29119527

[39] Byrne RM Pommier RF. Small Bowel and Colorectal Carcinoids. Clinics Colon Rectal Surg (2018) 31(5):301–8. 10.1055/s-0038-1642054 PMC6123011 30186052

[40] Vezzosi D Walter T Laplanche A Raoul JL Dromain C Ruszniewski P. Chromogranin A measurement in metastatic well-differentiated gastroenteropancreatic neuroendocrine carcinoma: screening for false positives and a prospective follow-up study. Int J Biol Markers (2011) 26(2):94–101. 10.5301/JBM.2011.8327 21574156

[41] Limbach KE Pommier SJ Dewey E Leon E Pommier RF. Neuroendocrine metastases to the ovaries are significantly associated with small bowel neuroendocrine tumors and carcinomatosis. Am J Surg (2020) 219(5):795–9. 10.1016/j.amjsurg.2020.02.040 32145918

[42] Hakim FA Alexander JA Huprich JE Grover M Enders FT. CT-enterography may identify small bowel tumors not detected by capsule endoscopy: eight years experience at Mayo Clinic Rochester. Digest Dis Sci (2011) 56(10):2914–9. 10.1007/s10620-011-1773-0 21735085

[43] Niederle B Pape UF Costa F Gross D Kelestimur F Knigge U. ENETS Consensus Guidelines Update for Neuroendocrine Neoplasms of the Jejunum and Ileum. Neuroendocrinology (2016) 103(2):125–38. 10.1159/000443170 26758972

[44] Massimino KP Han E Pommier SJ Pommier RF. Laparoscopic surgical exploration is an effective strategy for locating occult primary neuroendocrine tumors. Am J Surg (2012) 203(5):628–31. 10.1016/j.amjsurg.2011.12.010 22459446

[45] Bergsland EK Nakakura EK. Neuroendocrine tumors of unknown primary: is the primary site really not known? JAMA Surg (2014) 149(9):889–90. 10.1001/jamasurg.2014.216 25029597

[46] Hope TA Bergsland EK Bozkurt MF Graham M Heaney AP Herrmann K. Appropriate Use Criteria for Somatostatin Receptor PET Imaging in Neuroendocrine Tumors. J Nucl Med (2018) 59(1):66–74. 10.2967/jnumed.117.202275 29025982 PMC6910630

[47] Sadowski SM Neychev V Millo C Shih J Nilubol N Herscovitch P. Prospective Study of 68Ga-DOTATATE Positron Emission Tomography/Computed Tomography for Detecting Gastro-Entero-Pancreatic Neuroendocrine Tumors and Unknown Primary Sites. J Clin Oncol (2016) 34(6):588–96. 10.1200/JCO.2015.64.0987 PMC4872030 26712231

[48] Pavel M Oberg K Falconi M Krenning EP Sundin A Perren A. Gastroenteropancreatic neuroendocrine neoplasms: ESMO Clinical Practice Guidelines for diagnosis, treatment and follow-up. Ann Oncol Off J Eur Soc Med Oncol (2020) 31(7):844–60. 10.1016/j.annonc.2020.03.304 32272208

[49] Koea J. Commonwealth Neuroendocrine Tumour Research Collaborative Surgical S. Management of Locally Advanced and Unresectable Small Bowel Neuroendocrine Tumours. World J Surg (2020) 45(1):219–24. 10.1007/s00268-020-05740-7 32860138

[50] Zaidi MY Lopez-Aguiar AG Dillhoff M Beal E Poultsides G Makris E. Prognostic Role of Lymph Node Positivity and Number of Lymph Nodes Needed for Accurately Staging Small-Bowel Neuroendocrine Tumors. JAMA Surg (2019) 154(2):134–40. 10.1001/jamasurg.2018.3865 PMC6439661 30383112

[51] Wonn SM Limbach KE Pommier SJ Ratzlaff AN Leon EJ McCully BH. Outcomes of cytoreductive operations for peritoneal carcinomatosis with or without liver cytoreduction in patients with small bowel neuroendocrine tumors. Surgery (2020) 169(1):168–74. 10.1016/j.surg.2020.03.030 32473829

[52] Liu EH Solorzano CC Katznelson L Vinik AI Wong R Randolph G. AACE/ACE disease state clinical review: diagnosis and management of midgut carcinoids. Endocrine Pract Off J Am Coll Endocrinol Am Assoc Clin Endocrinologists (2015) 21(5):534–45. 10.4158/EP14464.DSC PMC4987303 25962092

[53] Norlen O Stalberg P Oberg K Eriksson J Hedberg J Hessman O. Long-term results of surgery for small intestinal neuroendocrine tumors at a tertiary referral center. World J Surg (2012) 36(6):1419–31. 10.1007/s00268-011-1296-z 21984144

[54] Ahmed A Turner G King B Jones L Culliford D McCance D. Midgut neuroendocrine tumours with liver metastases: results of the UKINETS study. Endocrine Related Cancer (2009) 16(3):885–94. 10.1677/ERC-09-0042 19458024

[55] Polcz M Schlegel C Edwards GC Wang F Tan M Kiernan C. Primary Tumor Resection Offers Survival Benefit in Patients with Metastatic Midgut Neuroendocrine Tumors. Ann Surg Oncol (2020) 27(8):2795–803. 10.1245/s10434-020-08602-7 PMC10184500 32430752

[56] Givi B Pommier SJ Thompson AK Diggs BS Pommier RF. Operative resection of primary carcinoid neoplasms in patients with liver metastases yields significantly better survival. Surgery (2006) 140(6):891–7; discussion 7-8. 10.1016/j.surg.2006.07.033 17188135

[57] Hellman P Lundstrom T Ohrvall U Eriksson B Skogseid B Oberg K. Effect of surgery on the outcome of midgut carcinoid disease with lymph node and liver metastases. World J Surg (2002) 26(8):991–7. 10.1007/s00268-002-6630-z 12016480

[58] Tsilimigras DI Ntanasis-Stathopoulos I Kostakis ID Moris D Schizas D Cloyd JM. Is Resection of Primary Midgut Neuroendocrine Tumors in Patients with Unresectable Metastatic Liver Disease Justified? A Systematic Review and Meta-Analysis. J Gastrointest Surg Off J Soc Surg Aliment Tract (2019) 23(5):1044–54. 10.1007/s11605-018-04094-9 30671800

[59] Condron ME Jameson NE Limbach KE Bingham AE Sera VA Anderson RB. A prospective study of the pathophysiology of carcinoid crisis. Surgery (2019) 165(1):158–65. 10.1016/j.surg.2018.04.093 30415870

[60] Rinke A Muller HH Schade-Brittinger C Klose KJ Barth P Wied M. Placebo-controlled, double-blind, prospective, randomized study on the effect of octreotide LAR in the control of tumor growth in patients with metastatic neuroendocrine midgut tumors: a report from the PROMID Study Group. J Clin Oncol (2009) 27(28):4656–63. 10.1200/JCO.2009.22.8510 19704057

[61] Caplin ME Pavel M Cwikla JB Phan AT Raderer M Sedlackova E. Anti-tumour effects of lanreotide for pancreatic and intestinal neuroendocrine tumours: the CLARINET open-label extension study. Endocrine Related Cancer (2016) 23(3):191–9. 10.1530/ERC-15-0490 PMC4740728 26743120

[62] Yao JC Fazio N Singh S Buzzoni R Carnaghi C Wolin E. Everolimus for the treatment of advanced, non-functional neuroendocrine tumours of the lung or gastrointestinal tract (RADIANT-4): a randomised, placebo-controlled, phase 3 study. Lancet (2016) 387(10022):968–77. 10.1016/S0140-6736(15)00817-X PMC6063317 26703889

[63] Strosberg J El-Haddad G Wolin E Hendifar A Yao J Chasen B. Phase 3 Trial of (177)Lu-Dotatate for Midgut Neuroendocrine Tumors. New Engl J Med (2017) 376(2):125–35. 10.1056/NEJMoa1607427 PMC5895095 28076709

[64] Vaghaiwalla T Ruhle B Memeh K Angelos P Kaplan E Liao CY. Response rates in metastatic neuroendocrine tumors receiving peptide receptor radionuclide therapy and implications for future treatment strategies. Surgery (2021) 169(1):162–7. 10.1016/j.surg.2020.04.001 32446596

[65] Network NCC. Neuroendocrine and Adrenal Tumors (Version 2.2020). (2020). Available at: https://www.nccn.org/professionals/physician_gls/pdf/neuroendocrine_blocks.pdf.

